# MCCMF: collaborative matrix factorization based on matrix completion for predicting miRNA-disease associations

**DOI:** 10.1186/s12859-020-03799-6

**Published:** 2020-10-14

**Authors:** Tian-Ru Wu, Meng-Meng Yin, Cui-Na Jiao, Ying-Lian Gao, Xiang-Zhen Kong, Jin-Xing Liu

**Affiliations:** School of Computer Science, Qufu Normal University, Rizhao, 276826 China

**Keywords:** MiRNA-disease association prediction, Matrix completion, Weight K Nearest Known Neighbors, Matrix factorization

## Abstract

**Background:**

MicroRNAs (miRNAs) are non-coding RNAs with regulatory functions. Many studies have shown that miRNAs are closely associated with human diseases. Among the methods to explore the relationship between the miRNA and the disease, traditional methods are time-consuming and the accuracy needs to be improved. In view of the shortcoming of previous models, a method, collaborative matrix factorization based on matrix completion (MCCMF) is proposed to predict the unknown miRNA-disease associations.

**Results:**

The complete matrix of the miRNA and the disease is obtained by matrix completion. Moreover, Gaussian Interaction Profile kernel is added to the miRNA functional similarity matrix and the disease semantic similarity matrix. Then the Weight K Nearest Known Neighbors method is used to pretreat the association matrix, so the model is close to the reality. Finally, collaborative matrix factorization method is applied to obtain the prediction results. Therefore, the MCCMF obtains a satisfactory result in the fivefold cross-validation, with an AUC of 0.9569 (0.0005).

**Conclusions:**

The AUC value of MCCMF is higher than other advanced methods in the fivefold cross validation experiment. In order to comprehensively evaluate the performance of MCCMF, accuracy, precision, recall and f-measure are also added. The final experimental results demonstrate that MCCMF outperforms other methods in predicting miRNA-disease associations. In the end, the effectiveness and practicability of MCCMF are further verified by researching three specific diseases.

## Background

MicroRNAs (MiRNAs) are a class of non-coding single-stranded RNA molecules. Their lengths are usually 18–24 nucleotides. Instead of synthesizing proteins, miRNAs participate in post-transcriptional regulation of gene expression in eukaryotes and viruses [1]. In spite of the first miRNA Line-4 was discovered in 1993 [2], the diversity and prevalence of these genes were revealed in recent years. To date, 38,589 miRNA have been found in animals, plants and viruses [3]. At the same time, miRNAs were discovered to play an important role in cell proliferation [4], differentiation [5], senescence [6], apoptosis [7], and so on. A study indicated that more than one third of human genes are regulated by miRNA [8]. Obviously, miRNA disorder could have severe impacts on humans.

Evidence shows that an increasing number of miRNAs are closely associated with diseases [9]. Since the first discovery of miR15 and miR16 deficiency in B cell chronic lymphocytic leukemia (B-CLL) [10], the research results of miRNA-disease associations are often reported. For example, the expression of miR-25 and miR-223 is significantly higher in patients with esophageal squamous cell carcinoma than the normal people, while the expression of miR-375 is significantly lower [11]. Studies show that miR-26a may be a regulatory factor that inhibits the progression and metastasis of c-Myc/EZH2 double height advanced HCC [12]. In addition, miR-340 has been suggested as a biomarker for cancer metastasis and prognosis [13]. At present, the research on miRNAs and diseases is becoming more extensive. Researchers have also developed a number of databases to store miRNA and disease data, such as dbDEMC [14], HMDD v3.0 [15] and miR2Disease [16]. Unfortunately, the known correlation data is not complete. Moreover, traditional methods to identify new miRNA-disease associations are time-consuming and laborious.

With the improvement of information technology and the development of a large number of miRNA data sets, many effective methods for predicting miRNA-disease associations have been proposed [17]. According to the hypothesis that functionally similar miRNAs may be associated with diseases with similar phenotypes [18], Jiang et al. [19] first constructed a genetic data network, and then prioritized disease-related miRNAs to predict miRNA-disease associations. However, due to the limited association information, this method is not quite effective. A computational framework was developed by Li et al., which can be used to measure the association between the cancer and miRNA based on the functional consistency score (FCS) of miRNA target genes and cancer-related genes. This method has a significant advantage in the identification of cancer-related miRNA [20]. Based on heterogeneous omics data, the potential miRNA-disease associations were identified via using a Graph Regularized Non-negative Matrix Factorization (GRNMF) by Xiao et al. [21]. However, the prediction results of GRNMF method may not be optimal in some cases. Chen et al. [22] proposed a new a computational model of Matrix Decomposition and Heterogeneous Graph Inference for miRNA-disease association prediction (MDHGI) to discover new miRNA-disease associations. The model made full use of matrix decomposition before the construction of heterogeneous networks, thus improving the prediction accuracy. The protein-driven inference of miRNA-disease associations (miRPD) was proposed by Mørk et al. [23], which can infer the correlation between miRNA-protein-disease associations. At the same time, they provide scoring schemes that can create correlation sets of high and medium credibility. Three new miRNA-disease association prediction methods based on global network similarity measure were developed by Chen et al. [24], namely MBSI (microRNA-based similarity inference), PBSI (phenotype-based similarity inference) and NetCBI (network-consistency-based inference). NetCBI is especially suitable for predicting target diseases, but it relies on network similarity measurement to a great extent. Similarly, Gao et al*.* [25] put forward a method based on Double Network Sparse Graph Regularized Matrix Factorization (DNSGRMF), and added the $L_{2,1}$-norm and Gaussian interaction profile (GIP) kernel to improve the prediction ability. In addition, considering the nearest neighbor information of the miRNA and the disease, Gao et al*.* [26] introduced a method of Nearest Profile-based Collaborative Matrix Factorization (NPCMF) to predict miRNA-disease associations. One of the most obvious disadvantages of NPCMF is that it introduces too much NP information, which may reduce the prediction accuracy while adding extra noise. In order to protect the known correlation, Logistic Weighted Profile-based Collaborative Matrix Factorization (LWPCMF) method was proposed by Yin et al*.* [27], which effectively predicts miRNA-disease associations. The prediction effect of this method is promising. Chen et al. [28] constructed a model based on Canonical Correlation analysis (CCA), which can fully reveal the possible molecular causes of miRNA-disease association. However, direct performance comparison is difficult to be achieved by this method.

In recent years, machine learning-based miRNA-disease association prediction methods are also popular. A support vector machine (SVM) classifier was developed by Xu et al. [29] to extract features from the miRNA-disease network and miRNA expression levels. Yet, the construction of miRNA target-dysregulated network (MTDN) is complex, so only direct miRNA target regulation can be predicted. Chen et al. [30] used random walk to prioritize disease-related miRNAs to predict potential human miRNA-disease associations. Like the problem of Jiang et al., their approach is also affected by limited disease-miRNA associations. A model of Restricted Boltzmann machine for multiple types of miRNA-disease association prediction (RBMMMDA) was established by Chen et al*.* [31]. Chen et al. [32] constructed a computational model called Laplacian Regularized Sparse Subspace Learning for MiRNA-Disease Association prediction (LRSSLMDA). The model has stronger dimensionality reduction capability and can be easily extended to higher dimensional data sets. A new Induction Matrix Completion model for MiRNA-Disease Association prediction (IMCMDA) was constructed by Chen et al. [33]. Because it is a semi-supervised model, only positive samples and unmarked samples are needed, which greatly reduces the difficulty of modeling. Soon after, Chen et al. [34] proposed a new MiRNA-Disease Association Prediction Bipartite graph Network Projection computing model (BNPMDA). Compared with previous models, the prediction accuracy of BNPMDA is improved. A new miRNA-disease association prediction algorithm based on the decision tree was proposed by Chen et al*.* [35]. This method constructs a computing framework for integrated learning and dimension reduction. By training and integrating multiple base classifiers, they reduce prediction bias and improve prediction performance. Ding et al. [36] used an improved calculation method based on inductive matrix completion to predict miRNA-disease associations. (IIMCMP). Experiments show that IIMCMP can achieve powerful and reliable performance evaluation. Li et al. [37] developed a method of neural inductive matrix completion with graph convolutional network (NIMCGCN) for the prediction of miRNA-disease association. To test the predictive power of NIMCGCN in the absence of any known miRNAs, they studied breast cancer with 100% accuracy. The above methods have made great contributions to predicting associations of miRNA-disease.

Since the shortcomings of the above methods, a novel method for predicting miRNA-disease associations with Collaborative Matrix Factorization based on Matrix Completion (MCCMF) is proposed in this paper. Firstly, human miRNA-disease association matrix, miRNA function similarity matrix and disease semantic similarity matrix are obtained from HMDD v2.0, but the obtained matrix is sparse. Therefore, the matrix completion method is used to complete the matrix. The matrix completion algorithm is mainly developed on the basis of Augmented Lagrange multiplier method (ALM) [38], Alternating Direction Method (ADM) [39] and Singular Value Threshold (SVT) operation [40]. Secondly, we integrate the completed matrix and the GIP kernel similarity matrix of the disease and the miRNA. At the same time, the miRNA-disease association matrix is preprocessed by Weight K Nearest Known Neighbors (WKNKN) method to solve the problem of unknown missing values [41]. Finally, collaborative matrix factorization is used to predict associations between miRNAs and diseases. In the experiment, a fivefold cross validation on MCCMF is performed, and results show that our method is superior to the other four methods. In addition, we focus on the cases of Gastrointestinal Neoplasms, Retinoblastoma and Hepatoblastoma. Our method not only successfully verifies the known associations of miRNA-disease, but also finds many unknown associations. To sum up, MCCMF can avoid the inherent noise of the data set, with high-speed and high prediction accuracy.

## Results

### Performance evaluation

In this section, AUC value, accuracy, precision, recall and f-measure are used to evaluate the performance of MCCMF method. Initially, we implement fivefold cross validation to objectively evaluate the predictive power of our method. The existing miRNA-disease associations are randomly divided into five groups, among which four groups are used as the training set and the remaining one as the test set. In addition, in order to demonstrate the high predictive capability of our method, the random deletion of the miRNA-disease association (i.e. Cross Validation pairs’ mode) increases the difficulty of prediction before performing the cross validation [42]. Fivefold cross validation is repeated 10 times to prevent grouping from causing bias, and the average result of 10 times is used as the final evaluation result.

The ROC curve is drawn to represent the predicted performance intuitively, and the AUC value is calculated to evaluate MCCMF quantitatively. TPR and FPR can be expressed as:

1 $$TPR = \frac{TP}{{TP + FN}},$$

2 $$FPR = \frac{FP}{{TN + FP}},$$

where $TP$ is the number of samples that are actually positive and are also predicted to be positive. $FN$ represents the number of samples that are actually negative and also predicted to be negative. However, $TN$ and $FP$ represent the number of samples for which the predicted results are inconsistent.

In order to make the performance evaluation more comprehensive, we also use other evaluation indicators, including the accuracy, precision, recall and f-measure. Their calculation formulas are defined as follows:

3 $$accuracy = \frac{TP + TN}{{TP + TN + FP + FN}},$$

4 $$precision = \frac{TP}{{TP + FP}},$$

5 $$recall = \frac{TP}{{TP + FN}},$$

6 $$f - measure = \frac{2 \times precision \times recall}{{precision \times recall}}.$$

### Comparison with other methods

**Fig. 1:**
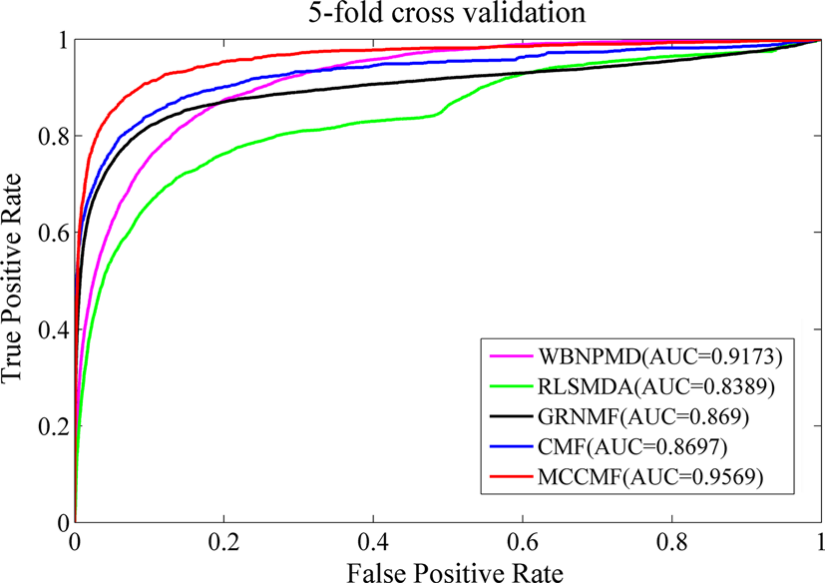
The ROC curves for each method in the fivefold cross validation experiment

The AUC value is generally between 0 and 1. The higher the AUC value is, the better the prediction result will be. MCCMF finally obtains an AUC value of 0.9563 in the fivefold cross validation. MCCMF is compared with four advanced methods such as WBNPMD [43], RLSMDA [44], GRNMF [21] and CMF [45], which proves the superior performance of our method. The ROC curves are drawn in Fig. 1, and the comparison results are listed in Table 1. The results of other methods in Table 1 are obtained directly from the literature.

**Table 1:** AUC results of cross validation experiments

Methods	Gold standard dataset
RLSMDA	0.8389 (0.0006)
GRNMF	0.869 (0.00023)
CMF	0.8697 (0.0011)
WBNPMD	0.9173 (0.0005)
*MCCMF*	*0.9569 (0.0005)*

In the Table 1, the highest value is highlighted in italic, with the standard deviation in parentheses. In the fivefold cross validation experiment, WBNPMD, RLSMDA, GRNMF, CMF and MCCMF obtain AUCs of 0.9173, 0.8389, 0.869, 0.8697 and 0.9569, respectively. Therefore, our method is superior to the other four methods.

**Fig. 2:**
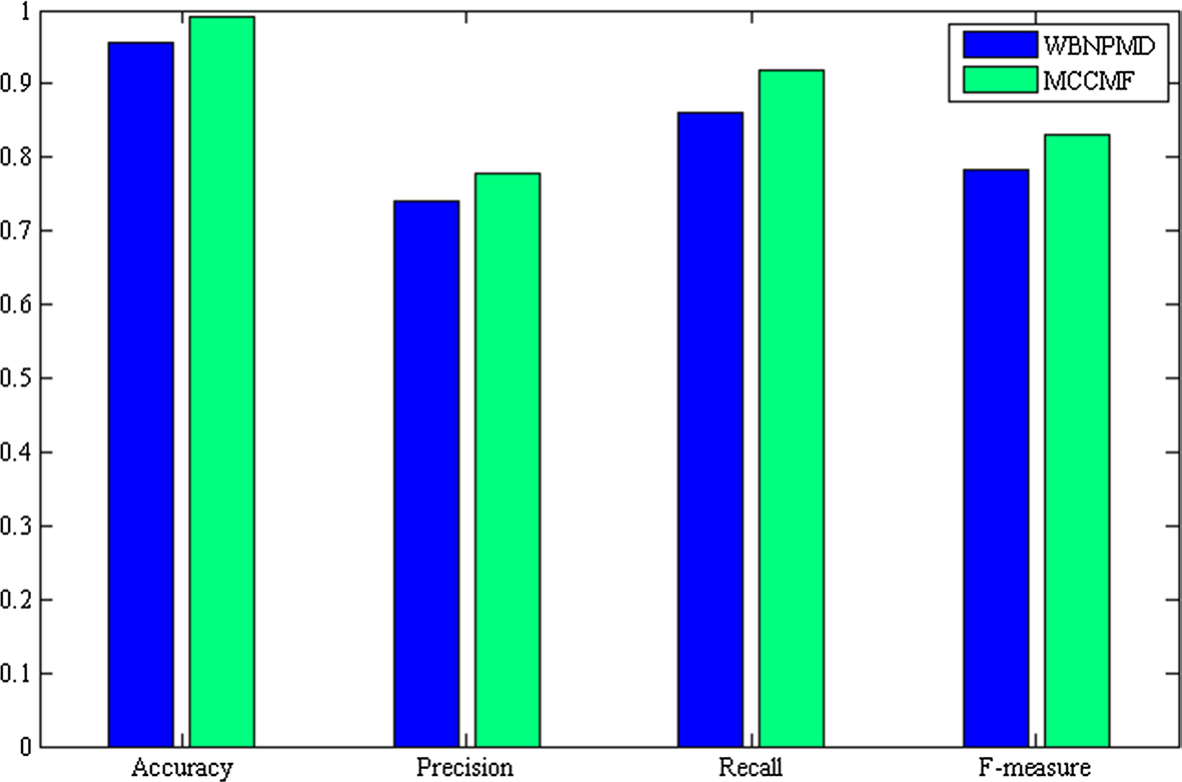
Comparison of the accuracy, precision, recall and f-measure with WBNPMD

WBNPMD with higher AUC value is selected for comparison with MCCMF, and accuracy, precision, recall and f-measure are presented as a bar graph in Fig. 2. Also, MCCMF is better than WBNPMD.

### Case studies

In the end, we carry out a simulation experiment to analyze the specific disease. First of all, the disease we want to explore is selected and the predicted score is ranked. Then, based on the predicted score after ranking, some miRNAs of high associations degree with the disease are found. Moreover, by comparing with the original miRNA-disease association matrix, they are determined whether the associations of high prediction score is known. Finally, the unknown associations are verified by searching existing data sets. Here, we choose three diseases of Gastrointestinal Neoplasms, Retinoblastoma and Hepatoblastoma for analysis. In addition, three popular data sets, dbDEMC [14], HMDD v3.0 [15] and miRCancer [46] are used for validation. These data sets store miRNA-disease associations that have been experimentally confirmed by some researchers over the years.

Gastrointestinal Neoplasms is a very common gastrointestinal disease with a high incidence. However, there are no obvious symptoms in the early growth stage of the neoplasms, which is very dangerous to human beings. We successfully predict 31 known associations and 9 new associations, 7 of which are confirmed by HMDD v3.0 and miRCancer. For example, Tazawa et al. [47] discovered the potential role of oncogenic miR-21 in Gastrointestinal Neoplasms. Other confirmed miRNAs have been reported in relevant data sets, and they are not listed here. There are still two unconfirmed ones that need further research. Table 2 describes the simulation results, where known associations are shown in italic, confirmed new predictions are written to the corresponding database, and unconfirmed ones are shown as “unconfirmed”. The predicted scores in the Table 2 are ranked according to the strength of the association between the miRNA and disease. There is a threshold to determine whether the prediction is accurate. Compared with known information and other databases, the prediction results of our method are generally accurate. Although two remain unconfirmed, these two could provide some insights for researchers.

**Table 2:** Predicted miRNAs for Gastrointestinal Neoplasms

Rank	MiRNA	Evidence	Rank	MiRNA	Evidence
1	has-mir-1	*Known*	21	has-let-7a	*Known*
2	has-mir-22	*Known*	22	has-mir-152	*Known*
3	has-mir-200	*Known*	23	has-mir-497	*Known*
4	has-mir-9	*Known*	24	has-mir-21	HMDD v3.0
5	has-mir-221	*Known*	25	has-mir-375	*Known*
6	has-mir-146a	*Known*	26	has-mir-107	*Known*
7	has-mir-133b	*Known*	27	has-mir-18b	*Known*
8	has-mir-200c	*Known*	28	has-mir-494	*Known*
9	has-mir-200a	*Known*	29	has-mir-150	miRCancer
10	has-mir-7	*Known*	30	has-mir-208a	*Known*
11	has-mir-200b	*Known*	31	has-mir-98	*Known*
12	has-mir-222	*Known*	32	has-mir-141	miRCancer
13	has-mir-126	*Known*	33	has-let-7g	*Known*
14	has-mir-196a	*Known*	34	has-mir-184	Unconfirmed
15	has-mir-142	*Known*	35	has-mir-210	miRCancer
16	has-mir-124	*Known*	36	has-mir-486	HMDD v3.0
17	has-mir-148a	*Known*	37	has-mir-338	*Known*
18	has-mir-451a	*Known*	38	has-mir-27a	miRCancer
19	has-mir-31	*Known*	39	has-mir-146b	HMDD v3.0
20	has-mir-451	*Known*	40	has-let-7c	Unconfirmed

Retinoblastoma is a malignant tumor that occurs in children under 3 years old, and has a familial predisposition. There are 38 known associations between the disease and miRNA in the known association data set, and 37 known associations are successfully predicted by us. At the same time, 23 new associations are predicted, seven of which are confirmed and the others are unconfirmed. Montoya et al. [48] found that the expression of miR-31 in Retinoblastoma is significantly reduced, which promotes the development of targeted therapy for Retinoblastoma. Table 3 shows the specific situation. The predictive sorting method in Table 3 is the same as that in Table 2.

**Table 3:** Predicted microbes for Retinoblastoma

Rank	MiRNA	Evidence	Rank	MiRNA	Evidence
1	has-mir-1	*Known*	31	has-mir-32	Unconfirmed
2	has-mir-9	*Known*	32	has-mir-200	Unconfirmed
3	has-mir-17	*Known*	33	has-mir-192	*Known*
4	has-mir-20a	*Known*	34	has-mir-513b	*Known*
5	has-mir-18a	*Known*	35	has-mir-135b	*Known*
6	has-mir-29c	*Known*	36	has-mir-513c	*Known*
7	has-mir-92a	*Known*	37	has-mir-22	miRCancer
8	has-let-7d	*Known*	38	has-mir-31	HMDD v3.0
9	has-let-7f	*Known*	39	has-mir-513a	*Known*
10	has-let-7g	*Known*	40	has-mir-30c	*Known*
11	has-let-7a	*Known*	41	has-mir-491	*Known*
12	has-mir-19b	*Known*	42	has-mir-135a	Unconfirmed
13	has-let-7b	*Known*	43	has-mir-125b	HMDD v3.0
14	has-mir-29b	*Known*	44	has-mir-7	Unconfirmed
15	has-let-7e	*Known*	45	has-mir-181a	Unconfirmed
16	has-let-7i	*Known*	46	has-mir-223	Unconfirmed
17	has-mir-124	*Known*	47	has-mir-210	Unconfirmed
18	has-let-7c	*Known*	48	has-mir-376a	*Known*
19	has-mir-19a	*Known*	49	has-mir-30a	Unconfirmed
20	has-mir-92	*Known*	50	has-mir-145	miRCancer
21	has-mir-181b	*Known*	51	has-mir-34b	HMDD v3.0
22	has-mir-34a	*Known*	52	has-mir-155	Unconfirmed
23	has-mir-29a	*Known*	53	has-mir-133a	Unconfirmed
24	has-mir-181	*Known*	54	has-mir-137	Unconfirmed
25	has-mir-24	*Known*	55	has-mir-146b	Unconfirmed
26	has-mir-142	*Known*	56	has-mir-150	Unconfirmed
27	has-mir-10b	*Known*	57	has-mir-126	Unconfirmed
28	has-mir-34c	*Known*	58	has-mir-18b	Unconfirmed
29	has-mir-125a	*Known*	59	has-mir-221	miRCancer
30	has-mir-21	HMDDv3.0/miRCancer	60	has-mir-373	HMDD v3.0

Hepatoblastoma is the most common intraabdominal malignant tumor after neuroblastoma and nephroblastoma in childhood. In the existing miRNA-disease association data set, there are 8 known miRNA-disease associations, and all of them have been predicted. Besides, we predicted 12 new associations, seven of which are confirmed and 5 are not. We also find literatures confirming that miR-143 is a factor affecting Hepatoblastoma. The study of Zhang et al. [49] showed that blocking miR-143 could significantly inhibit local liver metastasis. Hepatoblastoma prediction results are shown in Table 4. The predictive sorting method in Table 4 is also the same as that in Tables 2 and 3.

**Table 4:** Predicted microbes for Hepatoblastoma

Rank	MiRNA	Evidence	Rank	MiRNA	Evidence
1	has-mir-1	*Known*	11	has-mir-31	Unconfirmed
2	has-mir-21	*Known*	12	has-mir-126	dbDEMC
3	has-mir-150	*Known*	13	has-mir-146a	HMDD v3.0
4	has-mir-199a	HMDD v3.0	14	has-mir-125b	Unconfirmed
5	has-mir-143	HMDD v3.0	15	has-mir-148a	*Known*
6	has-mir-145	*Known*	16	has-mir-22	dbDEMC
7	has-mir-199b	*Known*	17	has-mir-210	HMDD v3.0
8	has-mir-214	*Known*	18	has-mir-138	Unconfirmed
9	has-mir-125a	*Known*	19	has-mir-133a	Unconfirmed
10	has-mir-9	Unconfirmed	20	has-mir-122	HMDD v3.0

**Fig. 3:**
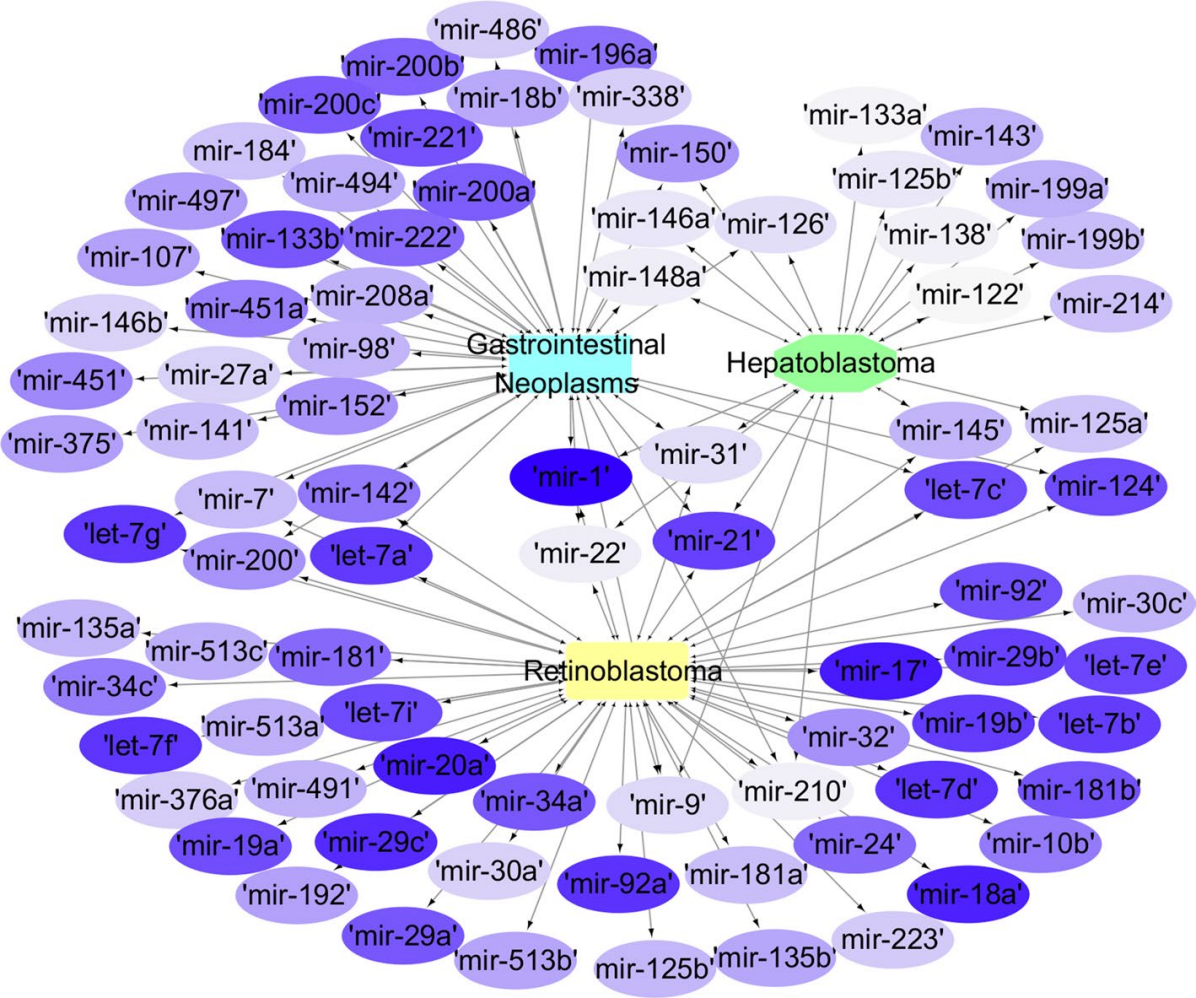
The association network between disease and miRNA

As can be seen from the simulation results above, most known miRNAs are successfully predicted, while a small number of unknown associations are in HMDDv3.0, miRCancer and dbDEMC data sets. Although a few have not been confirmed, they can be used as a reference for researchers. In addition, we used Cytoscape software to map the prediction network of these three diseases (Fig. 3). In the network, the ellipse represents miRNAs, and the remaining shapes represent diseases. The correlations are connected by line segments with arrows, and there are common miRNAs between diseases. According to the size of the predicted score, the color degree of the ellipse is set differently. The darker the color of the ellipse is set to, the stronger the correlation between miRNA and disease is.

## Discussion

The above experimental results are enough to prove that our method is superior to the most advanced method. The excellent prediction performance of MCCMF can be attributed to several significant factors. Firstly, data is preprocessed by Weight K Nearest Known Neighbors method and matrix completion method to improve the prediction accuracy. Secondly, a collaborative matrix factorization model is applied to predicting miRNA-disease associations, which is a promising one among many collaborative filtering technologies. In bioinformatics, matrix factorization contributes to identifying hidden links among genes. However, the performance of our method needs to be further improved. For instance, there exists a better way to integrate data, rather than simply adding them together. In the future, we will improve the technology to use the latest version of the data set, such as HMDD v3.0.

## Conclusions

In this paper, a collaborative matrix factorization method based on matrix completion (MCCMF) is developed for predicting miRNA-disease associations. Considering the sparse and incomplete similarity matrix of miRNA-disease, we use the matrix completion method to complete the matrix. Then the completed matrix is integrated with GIP kernel similarity to improve the data information and reduce the influence of noises. In addition, WKNKN is also introduced to pretreat the existing association matrix of miRNAs and diseases, so our method is suitable to practical problems. Finally, the idea of CMF is adopted to construct the objective function and obtain the predicted results. The AUC value (0.9569) of MCCMF is higher than other advanced methods in the fivefold cross validation experiment. In order to comprehensively evaluate the performance of MCCMF, accuracy, precision, recall and f-measure are applied to measure the performance, and results are 0.992, 0.779, 0.918 and 0.830, respectively. Compared with the other four methods, our method has the best performance. The analysis of Gastrointestinal Neoplasms, Retinoblastoma and Hepatoblastoma further verified the effectiveness of MCCMF. Since most of associations are unknown in reality, MCCMF can also be used to predict in this situation.

## Methods

**Fig. 4:**
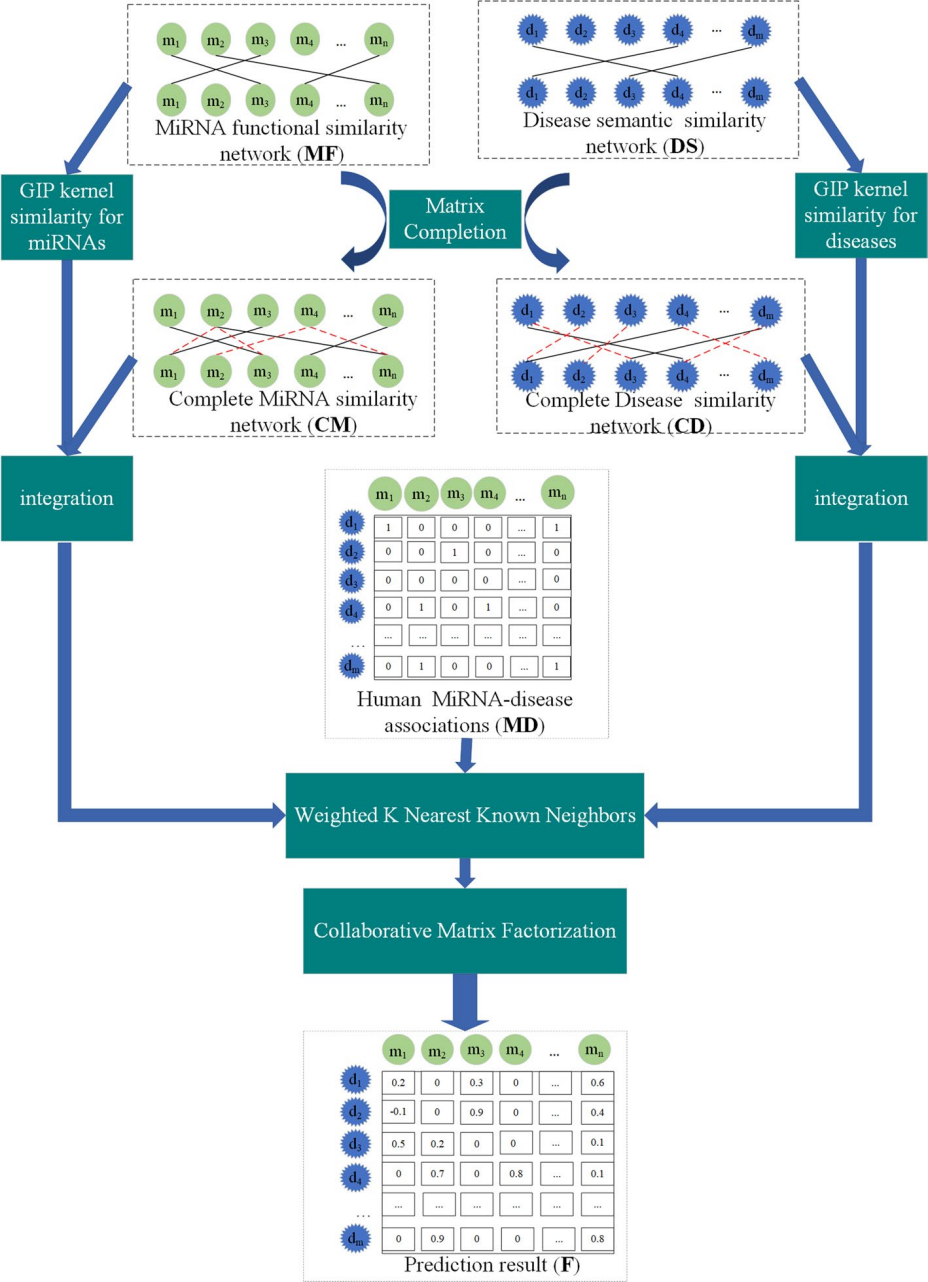
Flowchart of potential disease‐miRNA association prediction based on the model of MCCMF

We develop a novel method for predicting miRNA-disease associations with MCCMF. MCCMF is divided into four main steps: Firstly, we use the matrix completion algorithm to complete the miRNA similarity matrix and the disease similarity matrix to generate a new completion similarity matrix. Secondly, the new completion similarity matrix is integrated with existing miRNA and disease similarity information. Thirdly, the WKNKN is used to convert the binary values of the miRNA-disease association matrix into the interaction likelihood values [41]. Finally, the Collaborative Matrix Factorization is used to predict the association of miRNA-disease. Figure 4 shows the complete process for MCCMF.

### Human miRNA-disease associations

The initial miRNA-disease association data is downloaded from HMDD v2.0 [50]. HMDD v2.0 is an experimental data set supporting human miRNA-disease associations, and storing 5430 experimentally verified miRNA-disease associations between 495 miRNAs and 383 diseases. In this paper, the adjacency matrix ${\mathbf{MD}}$ is used to represent the miRNA-disease association network. The adjacency matrix ${\mathbf{MD}}$ is a sparse matrix composed of 0 and 1. If ${\mathbf{MD}}\left( {m_{i} ,d_{j} } \right)$ is 1, disease $d_{j}$ is correlated with miRNA $m_{i}$; otherwise irrelevant.

### MiRNA function similarity

According to the hypothesis that functionally similar miRNAs are more likely to be associated with phenotypic diseases, a method for calculating the functional similarity of miRNAs (MISIM) is proposed by Wang et al. [51]. Firstly, we need to define semantic similarity between one disease and one group of disease. The calculation formula is as follows:

7 $$S(d,{\mathbf{D}}) = \mathop {\max }\limits_{1 \le i \le k} (S(d,{\mathbf{D}}_{{\text{i}}} )).$$

Here $d$ represents one disease and ${\mathbf{D}}$ represents one disease group. Then, we define the similarity of $d$ and ${\mathbf{D}}$, $S(d,{\mathbf{D}})$, as the maximum similarity.

Functional similarity of the two miRNAs is defined as

8 $$MISIM(M_{1} ,M_{2} ) = \frac{{\sum\nolimits_{1 \le i \le m} {S(d_{1i} ,{\mathbf{D}}_{2} ) + \sum\nolimits_{1 \le j \le n} {S(d_{2i} ,{\mathbf{D}}_{1} )} } }}{m + n},$$

where $M_{1}$ and $M_{2}$ represent the related miRNAs of ${\mathbf{D}}_{1}$ and ${\mathbf{D}}_{2}$, respectively. ${\mathbf{D}}_{1}$ contains $m$ diseases, and ${\mathbf{D}}_{2}$ contains $n$ diseases.

In this paper, we download the miRNA function similarity from https://www.cuilab.cn/files/images/cuilab/misim.zip. And the matrix ${\mathbf{MF}}$ is used to represent the functional similarity network of the miRNA, in which the element ${\mathbf{MF}}(i,j)$ represents the similarity between miRNA $m_{i}$ and miRNA $m_{j}$. The self-similarity of each miRNA is 1, so the diagonal elements of the matrix ${\mathbf{MF}}$ are 1.

Due to incomplete miRNA data supported by the experiment, the similarity values calculated by MISIM may be biased. Some subsequent treatment of the matrix may be improved [52].

### Disease semantic similarity

The relationship between different diseases is obtained from the MeSH database (https://www.ncbi.nlm.nih.gov/). Based on the previous literature [51], we represent the disease $D$ as a Directed Acyclic Graph, $DAG(D) = (D,T(D),E(D))$, where $T(D)$ is the set of both a node $D$ and its ancestor nodes, and $E(D)$ is the set of edges that ancestor nodes pointing to node $D$. For ancestor node $t$ in $DAG(A)$, its contribution to the semantic value of disease $A$ is computed as follows:

9 $$D1_{A} (t) = \left\{ {\begin{array}{*{20}l} 1 \hfill & {if\,\,t = A,} \hfill \\ {\max \left\{ {\Delta *D1_{A} \left( {t^{^{\prime}} } \right)|t^{^{\prime}} \in children\,of\,t} \right\}} \hfill & {if\,\,t \ne A.} \hfill \\ \end{array} } \right.$$

In the above formula, $\Delta$ is a semantic contribution factor. Based on the method of Wang et al., the value of $\Delta$ is set to 0.5. For the disease $A$, the contribution of itself to the disease $A$ is 1, while the contribution of ancestor node $t$ is decreasing with the increase of its layers.

Based on the contribution of ancestor diseases and disease $A$ itself, the semantic value of disease $A$ can be expressed as follows:

10 $$DV1\left( A \right) = \sum\limits_{t \in T\left( A \right)} {D1_{A} \left( t \right)} .$$

According to the hypothesis that the more shared part of the disease pairs in $DAGs$ is, the higher similarity is. The semantic similarity between disease $A$ and disease $B$ is calculated as:

11 $$DS1\left( {A,B} \right) = \frac{{\sum\nolimits_{t \in T\left( A \right) \cap T\left( B \right)} {\left( {D1_{A} \left( t \right) + D1_{B} \left( t \right)} \right)} }}{DV1\left( A \right) + DV1\left( B \right)}.$$

However, the above model is a little inadequacy, which is the setting of $\Delta$ that causes the same layer of diseases with the same semantic contribution. Obviously, the incidence of various diseases is different, and the contribution of diseases with high incidence should be less than those with low incidence. To improve the above model, we combine the method of Xuan et al. [53] to define the semantic similarity calculation method. In this method, the contribution of ancestor node $t$ in $DAG(A)$ to the semantic value of disease $A$ is as follows:

12 $$D2_{A} \left( t \right) = - \log \frac{{the\,number\,of\,DAG{\text{s}} \,including\,t}}{the\,number\,of\,diseases}.$$

The semantic value of disease $A$, and the semantic similarity between the disease $A$ and the disease $B$ are calculated as:

13 $$DV2\left( A \right) = \sum\limits_{t \in T\left( A \right)} {D2_{A} \left( t \right)} ,$$

14 $$DS2\left( {A,B} \right) = \frac{{\sum\nolimits_{t \in T\left( A \right) \cap T\left( B \right)} {\left( {D2_{A} \left( t \right) + D2_{B} \left( t \right)} \right)} }}{DV2\left( A \right) + DV2\left( B \right)}.$$

Finally, in order to calculate the semantic similarity more comprehensive and rational, we combine the two models to get Eq. (15).

15 $${\mathbf{DS}}\left( {A,B} \right) = \frac{{DS1\left( {A,B} \right) + DS2\left( {A,B} \right)}}{2}.$$

### Gaussian interaction profile kernel similarity for diseases and miRNAs

On the basis of the hypothesis that functionally similar miRNAs may be associated with similar diseases, and vice versa, the known miRNA-disease association network is used to construct the GIP kernel similarity for diseases and miRNAs [54]. GIP kernel similarity can increase the multiple and topological information of known correlations. The interaction profile of miRNA $m(i)$ is represented by the binary vector $M(i)$ of the *i*-th column of the adjacency matrix ${\mathbf{MD}}$. Similarly, the binary vector $D(i)$ of the *i*-th row of the adjacency matrix ${\mathbf{MD}}$ denotes the interaction profile of disease $d(i)$. Hence, we can define the GIP kernel similarity for miRNAs and diseases as follows:

16 $${\mathbf{GM}}(m(i),m(j)) = \exp ( - \gamma_{m} ||M(i) - M(j)||^{2} ),$$

17 $${\mathbf{GD}}(d(i),d(j)) = \exp ( - \gamma_{d} ||D(i) - D(j)||^{2} ).$$

Here, $\gamma_{m}$ and $\gamma_{d}$ are parameters to control the kernel bandwidth and obtained by the following formulas:

18 $$\gamma_{m} = \frac{{\delta_{m} }}{{\frac{1}{nm}\sum\nolimits_{i = 1}^{nm} {||M(i)||^{2} } }},$$

19 $$\gamma_{d} = \frac{{\delta_{d} }}{{\frac{1}{nd}\sum\nolimits_{i = 1}^{nd} {||D(i)||^{2} } }},$$

where $\delta_{m}$ and $\delta_{d}$ are also bandwidth parameters and they are set to 1 according to the previous study [55]. The $nm$ and $nd$ mean the number of all the miRNAs and diseases.

### Matrix completion

The miRNA functional similarity matrix and disease semantic similarity matrix calculated by the above operations are still sparse and incomplete, and there are some redundant associations (i.e. inherent noise). So we use the matrix completion method to solve the problem [56]. Suppose the incomplete matrix is ${\mathbf{D}}$, which can be represented as a linear combination of ${\mathbf{D}}$ and the noise matrix ${\mathbf{N}}$. The formula is as follows:

20 $${\mathbf{D = DR + N}},$$

where ${\mathbf{DR}}$ is a low-rank matrix, and specifically, it is a more refined or informative similarity matrix after removing noise from the existing similarity matrix.

In order to make ${\mathbf{R}}$ be low-rank, a nuclear norm on ${\mathbf{D}}$ is added. At the same time, the $L_{2,1}$-norm of the error term ${\mathbf{N}}$ is used to make noise matrix ${\mathbf{N}}$ more sparse. When the final low-rank matrix ${\mathbf{DR}}^{*}$ and sparse matrix ${\mathbf{N}}^{*}$ are calculated, ${\mathbf{DR}}^{*}$ or ${\mathbf{D}} - {\mathbf{N}}^{*}$ are used to describe a completed matrix. Therefore, a formula for solving convex optimization problem can be defined as follows:

21 $$\mathop {\min }\limits_{{{\mathbf{R}},{\mathbf{N}}}} ||{\mathbf{R}}||_{*} + \omega ||{\mathbf{N}}||_{2,1} s.t.\,{\mathbf{D}} = {\mathbf{DR}} + {\mathbf{N}}.$$

Here, $|| \cdot ||_{*}$ represents the nuclear norm, $\omega \in (0,1)$ is the positive weighting parameter and $|| \cdot ||_{2,1}$ is the noise regularization term.

When solving optimization problems under equality constraints, the ALM method is more effective [38]. Therefore, according to ALM, the Eq. (21) can be rewritten as:

22 $$\mathop {\min }\limits_{{{\mathbf{R,N,X}}}} ||{\mathbf{X}}||_{*} + \omega ||{\mathbf{N}}||_{2,1} s.t.\,{\mathbf{D}} = {\mathbf{DR}} + {\mathbf{N}},{\mathbf{R}} = {\mathbf{X}}.$$

Then switch the Eq. (22) to an unconstraint problem, which is the Lagrange function. The formula is as follows:

23 $$\begin{aligned} L_{\beta } ({\mathbf{X}},{\mathbf{R}},{\mathbf{N}}) & = ||{\mathbf{X}}||_{*} + \omega ||{\mathbf{N}}||_{2,1} \\ & \quad + tr(Y_{1}^{T} ({\mathbf{D}} - {\mathbf{DR}} - {\mathbf{N}})) + tr(Y_{2}^{T} ({\mathbf{R}} - {\mathbf{X}})) \\ & \quad + \frac{\beta }{2}(||{\mathbf{D}} - {\mathbf{DR}} - {\mathbf{N}}||_{F}^{2} + ||{\mathbf{R}} - {\mathbf{X}}||_{F}^{2} ), \\ \end{aligned}$$

where $\beta > 0$ is the penalty parameter, and $\beta$ is updated by $\beta = \min (\rho \beta ,\max_{\beta } )$. $Y_{1}$ and $Y_{2}$ are the Lagrange multipliers.

The ADM method is used to solve the Eq. (23) [39]. The ADM is a simple method to solve the decomposable convex optimization problem, especially in solving large-scale problems. The update iterations for ADM are as follows:

24 $$\left\{ \begin{gathered} {\mathbf{X}}^{k + 1} = \arg \mathop {\min }\limits_{X} L({\mathbf{X}},{\mathbf{R}}^{k} ,{\mathbf{N}}^{k} ,\beta ), \hfill \\ {\mathbf{R}}^{k + 1} = \arg \mathop {\min }\limits_{R} L({\mathbf{X}}^{k + 1} ,{\mathbf{R}},{\mathbf{N}}^{k} ,\beta ), \hfill \\ {\mathbf{N}}^{k + 1} = \arg \mathop {\min }\limits_{N} L({\mathbf{X}}^{k + 1} ,{\mathbf{R}}^{k + 1} ,{\mathbf{N}},\beta ). \hfill \\ \end{gathered} \right.$$

Based on the singular value shrinkage operator [40], ${\mathbf{X}}^{k + 1}$ and ${\mathbf{N}}^{k + 1}$ are represented as follows:

25 $${\mathbf{X}}^{k + 1} = D_{{\frac{1}{\beta }}} \left( {{\mathbf{R}} + \frac{{Y_{2} }}{\beta }} \right) = argmin\frac{1}{\beta }||{\mathbf{X}}||_{*} + \frac{1}{2}\left\| {{\mathbf{X}} - \left( {{\mathbf{R}} + \frac{{Y_{2} }}{\beta }} \right)} \right\|_{F}^{2} ,$$

26 $${\mathbf{N}}^{k + 1} = D_{{\frac{\omega }{\beta }}} \left( {{\mathbf{D}} - {\mathbf{DR}} + \frac{{Y_{1} }}{\beta }} \right) = argmin\frac{w}{\beta }||{\mathbf{N}}||_{2,1} + \frac{1}{2}\left\| {{\mathbf{N}} - \left( {{\mathbf{D}} - {\mathbf{DR}} + \frac{{Y_{1} }}{\beta }} \right)} \right\|_{F}^{2} ,$$

yet the minimization of ${\mathbf{R}}$ is a least squares problem, and its normal equation is as follows:

27 $${\mathbf{R}} = ({\mathbf{I}} + {\mathbf{D}}^{T} {\mathbf{D}})^{ - 1} \left( {{\mathbf{D}}^{T} {\mathbf{D}} - {\mathbf{D}}{}^{T}{\mathbf{N}} + {\mathbf{X}} + \frac{{{\mathbf{D}}^{T} Y_{1} - Y_{2} }}{\beta }} \right),$$

where ${\mathbf{I}} = {\mathbf{DD}}^{T}$ is widely used in matrix completion.

Then ${\mathbf{X}}$, ${\mathbf{R}}$ and ${\mathbf{N}}$ are updated by changing the Lagrange multipliers $Y_{1}$ and $Y_{2}$. Moreover, $Y_{1}$ and $Y_{2}$ can be obtained by the following formulas:

28 $$Y_{1} = Y_{1} + \beta ({\mathbf{D}} - {\mathbf{DR}} - {\mathbf{E}}),$$

29 $$Y_{2} = Y_{2} + \beta ({\mathbf{R}} - {\mathbf{X}}).$$

**Fig. 5:**
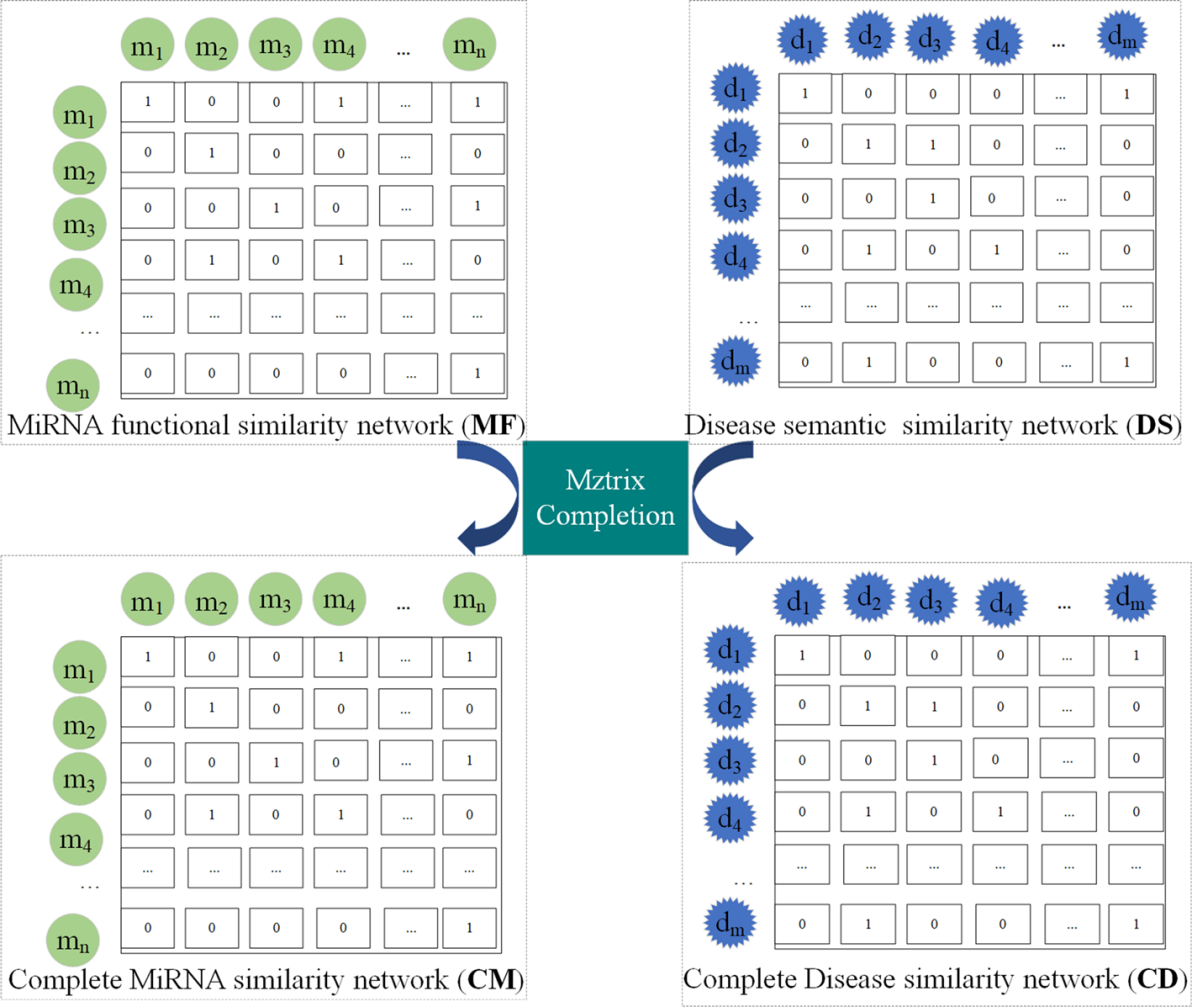
The process of matrix completion

Finally, we can get the final low-rank matrix ${\mathbf{R}}^{*}$ and sparse matrix ${\mathbf{N}}^{*}$ until the convergence conditions $||{\mathbf{D}} - {\mathbf{DR}} - {\mathbf{N}}||_{\infty } < \varepsilon$ and $||{\mathbf{R}} - {\mathbf{X}}||_{\infty } < \varepsilon$ are satisfied. Here, $\varepsilon$ is an extremely low number (set as $1 \times 10^{ - 8}$ in this paper). As mentioned above, the refined matrix ${\mathbf{R}}^{*}$ and noise matrix ${\mathbf{N}}^{*}$ can be used to describe a completed matrix in the form of ${\mathbf{D}} \times {\mathbf{R}}^{*}$ or ${\mathbf{D}} - {\mathbf{N}}^{*}$. The specific process of matrix completion is shown in Fig. 5.

Based on the above matrix completion method, the disease semantic similarity matrix ${\mathbf{DS}}$ and miRNA functional similarity matrix ${\mathbf{MF}}$ are used as input matrices to replace matrix ${\mathbf{D}}$, so that we can obtain two refined similarity matrices ${\mathbf{CD}}$ and ${\mathbf{CM}}$, respectively.

The algorithm of Matrix completion is summarized in Algorithm 1.
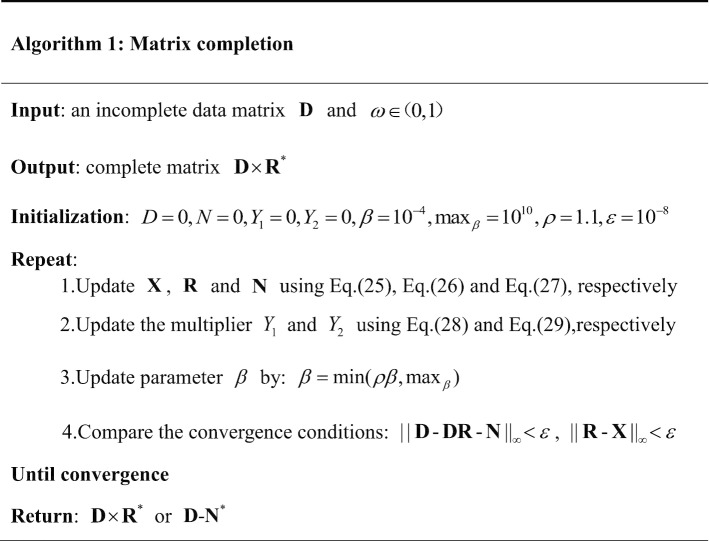


### Similarity information integrations

**Fig. 6:**
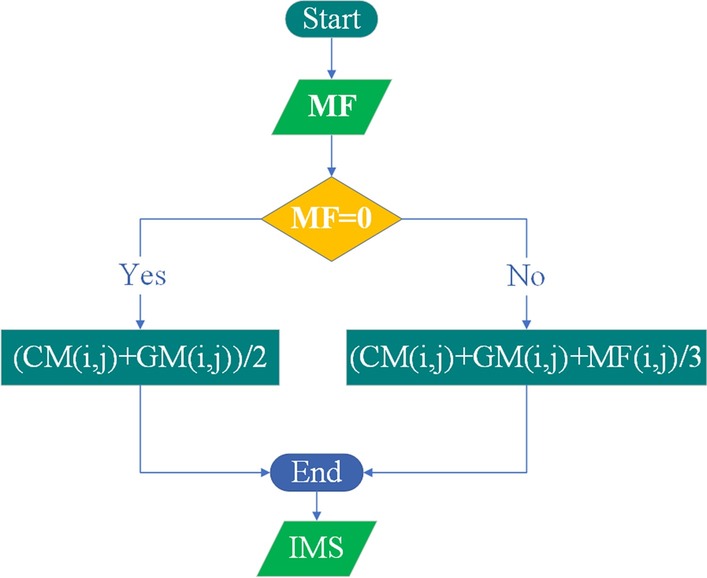
The flowchart of an integration method. Matrix MF is the miRNA functional similarity matrix and GM is the GIP kernel similarity for miRNAs and CM is the Complete MiRNA

Subsequent work is to integrate the completed matrix with existing similarity matrices. Since similarity information integrations of diseases and miRNAs are similar, Fig. 6 only shows the process for integration of miRNA similarity.

The specific integration formulas are as follows:

30 $${\mathbf{IMS}}(i,j) = \left\{ {\begin{array}{*{20}l} {\frac{{{\mathbf{CM}}(i,j) + {\mathbf{GM}}(i,j)}}{2}{,}} \hfill & {{\text{if}}\,\,{\mathbf{MF}}{\text{(i,j) = 0,}}} \hfill \\ {\frac{{{\mathbf{GM}}(i,j) + {\mathbf{CM}}(i,j) + {\mathbf{MF}}(i,j)}}{3},} \hfill & {\text{otherwise,}} \hfill \\ \end{array} } \right.$$

31 $${\mathbf{IDS}}(i,j) = \left\{ {\begin{array}{*{20}l} {\frac{{{\mathbf{CD}}(i,j) + {\mathbf{GD}}(i,j)}}{2}{,}} \hfill & {{\text{if}}\,{\mathbf{DS}}{\text{(i,j) = 0,}}} \hfill \\ {\frac{{{\mathbf{GD}}(i,j) + {\mathbf{CD}}(i,j) + {\mathbf{DS}}(i,j)}}{3},} \hfill & {\text{otherwise,}} \hfill \\ \end{array} } \right.$$

### WKNKN

WKNKN can be thought of as a voting or integration method: some potential classifiers (nearest neighbors) are aggregated by a (weight) majority vote, the results of which are used for prediction [41].

In this paper, ${\mathbf{MD}}$ expresses the miRNA-disease association matrix, which only represents the association between the miRNA and the disease verified by human experiment at the current stage. And we simply stipulate that if the miRNA is associated with the disease, ${\mathbf{MD}}\left( {m_{i} ,d_{j} } \right)$ will be set to 1. However, there are still many unknown miRNAs and diseases in the world, and whether they can be used as a bridge between existing miRNAs and diseases or not are still unknown. Maybe existing miRNAs are correlated with existing diseases through these unknown miRNAs, so the ${\mathbf{MD}}$ regulation is obviously inappropriate.

**Fig. 7:**
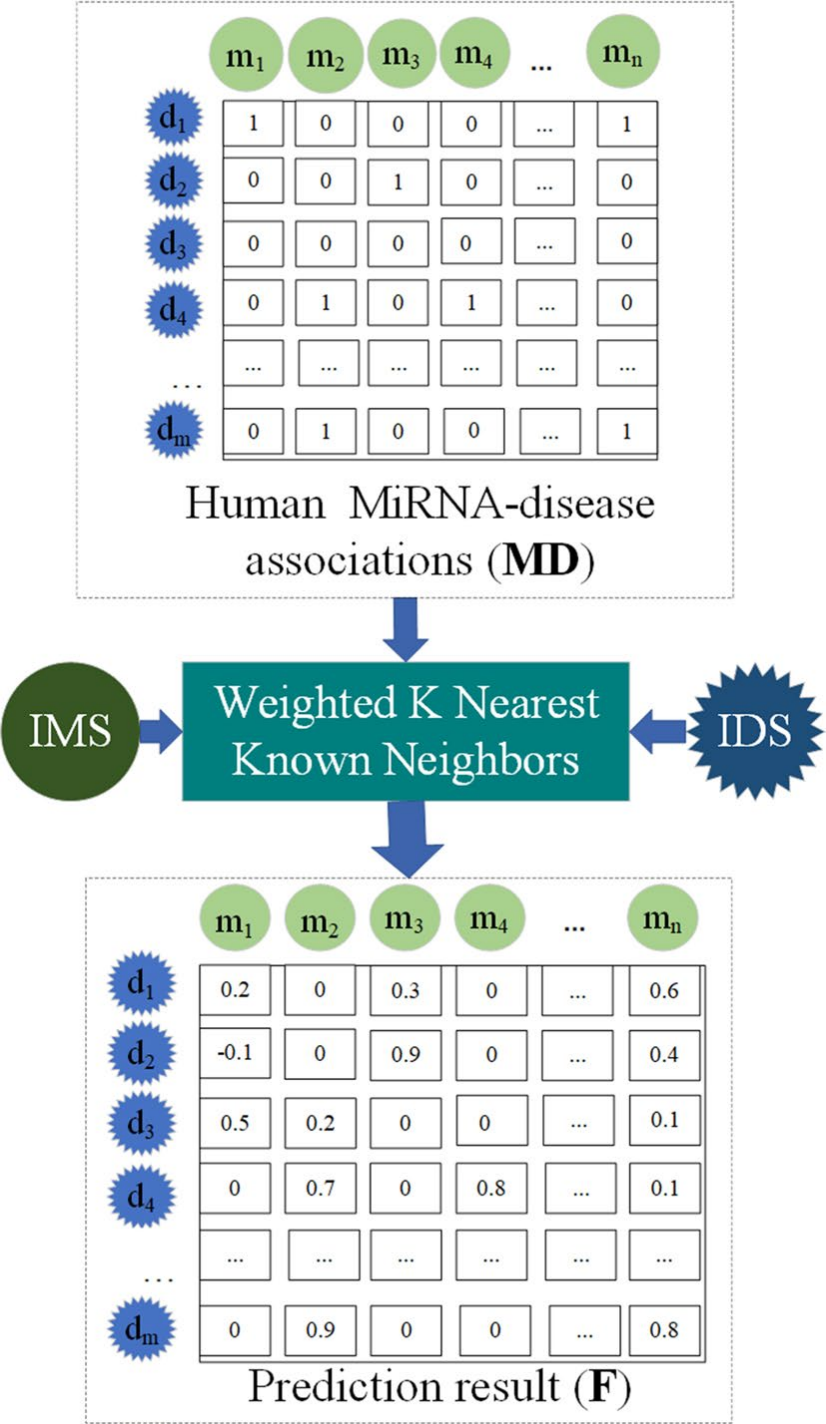
The process of WKNKN. IMS and IDS are similar integration matrices of miRNA and disease, respectively

Therefore, by estimating these unknown conditions through the correlation of its known neighbors, the WKNKN method preprocesses the matrix ${\mathbf{MD}}$ to get the pre-processed matrix of ${\mathbf{MD}}$ (${\mathbf{PMD}}$). If ${\mathbf{MD}}\left( {m_{i} ,d_{j} } \right) = 0$, WKNKN will give ${\mathbf{MD}}\left( {m_{i} ,d_{j} } \right)$ a value from 0 to 1 according to the corresponding similar information of miRNAs and diseases. The specific process of WKNKN is shown in Fig. 7.

### MCCMF for MiRNA-disease association prediction

The CMF method proposed by Shen et al. [45] that can effectively predict the potential interactions between miRNAs and diseases. In this study, the idea of the CMF method is used to predict the miRNA-disease association. The specific steps of CMF are as follows: firstly, the input miRNA-disease association matrix ${\mathbf{PMD}}$ is decomposed into two low-rank matrices ${\mathbf{A}}$ and ${\mathbf{B}}$ by using the singular value decomposition.

32 $$\begin{aligned} & [{\mathbf{U}},{\mathbf{S}},{\mathbf{V}}] = SVD({\mathbf{PMD}},k), \\ & {\mathbf{A}} = {\mathbf{US}}_{k}^{\frac{1}{2}} , \\ & {\mathbf{B}} = {\mathbf{VS}}_{k}^{\frac{1}{2}} , \\ \end{aligned}$$

where ${\mathbf{U}}$ and ${\mathbf{V}}$ is the unitary matrix. ${\mathbf{S}}$ is a negative real diagonal matrix, and there are k singular values on the diagonal.

Secondly, we write the objection function of MCCMF according to the idea of CMF, as follows:

33 $$\begin{aligned} & \min_{{{\mathbf{A}},{\mathbf{B}}}} ||{\mathbf{PMD}} - {\mathbf{AB}}^{T} ||_{F}^{2} + \lambda_{l} (||{\mathbf{A}}||_{F}^{2} + ||{\mathbf{B}}||_{F}^{2} ) \\ & \quad + \lambda_{m} ||{\mathbf{IMS}} - {\mathbf{AA}}^{T} ||_{F}^{2} \\ & \quad + \lambda_{d} ||{\mathbf{IDS}} - {\mathbf{BB}}^{T} ||_{F}^{2} . \\ \end{aligned}$$

Here, $|| \cdot ||_{F}$ is the Frobenius norm to ensure that the feature vectors of similar miRNAs and similar diseases are similar. $\lambda_{l}$, $\lambda_{m}$ and $\lambda_{d}$ are positive parameters, which are determined by the fivefold cross validation, and $\lambda_{l} \in \left\{ {2^{ - 2} ,2^{ - 1} ,2^{0} ,2^{1} } \right\}$, $\lambda_{m} /\lambda_{d} \in \left\{ {2^{ - 3} ,2^{ - 2} ,2^{ - 1} ,2^{0} ,2^{1} ,2^{2} ,2^{3} ,2^{4} ,2^{5} } \right\}$.

Thirdly, we use $L$ to represent the Eq. (33), and derive two alternative update rules by setting ${{\partial L} \mathord{\left/ {\vphantom {{\partial L} {\partial {\mathbf{A}}}}} \right. \kern-\nulldelimiterspace} {\partial {\mathbf{A}}}} = 0$ and ${{\partial L} \mathord{\left/ {\vphantom {{\partial L} {\partial {\mathbf{B}}}}} \right. \kern-\nulldelimiterspace} {\partial {\mathbf{B}}}} = 0$.

34 $${\mathbf{A}} = ({\mathbf{PMD}}*{\mathbf{B}} + \lambda_{{\text{m}}} {\mathbf{IMS}}*{\mathbf{A}})({\mathbf{B}}^{T} {\mathbf{B}} + \lambda_{l} {\mathbf{I}}_{k} + \lambda_{m} {\mathbf{A}}^{T} {\mathbf{A}})^{ - 1} ,$$

35 $${\mathbf{B}} = ({\mathbf{PMD}}^{T} *{\mathbf{A}} + \lambda_{{\text{d}}} {\mathbf{IDS}}*{\mathbf{B}})({\mathbf{A}}^{T} {\mathbf{A}} + \lambda_{l} {\mathbf{I}}_{k} + \lambda_{d} {\mathbf{B}}^{T} {\mathbf{B}})^{ - 1} ,$$

where ${\mathbf{I}}_{k}$ is the $k \times k$ identity matrix.

**Fig. 8:**
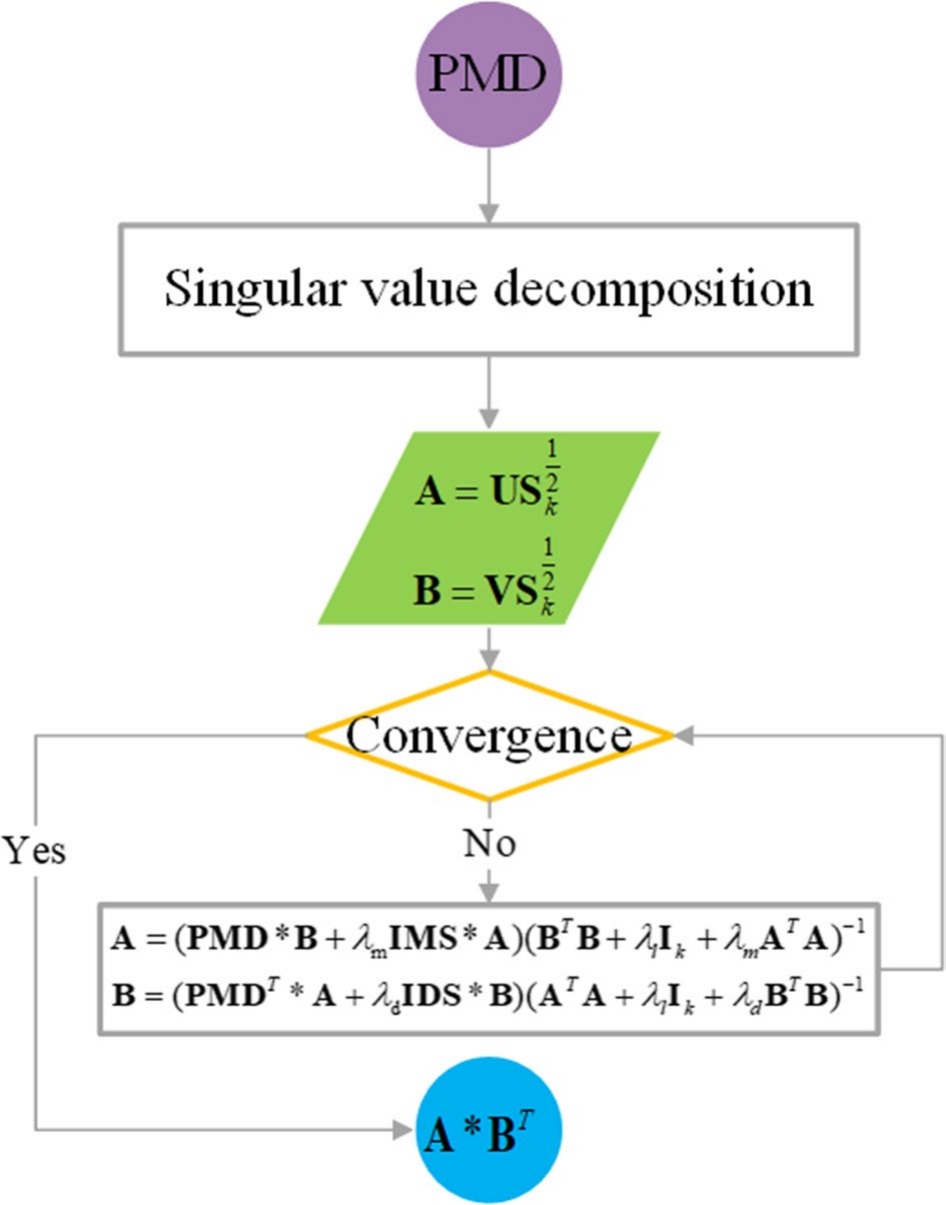
The flowchart of CMF method. PMD is the pre-processed matrix of miRNA-disease association matrix

Finally, we update ${\mathbf{A}}$ and ${\mathbf{B}}$ iteratively until they converge to get the final ${\mathbf{A}}$ and ${\mathbf{B}}$. By ${\mathbf{A}}*{\mathbf{B}}^{T}$, the prediction matrix for miRNA-disease associations is obtained. The detail process of MCCMF can be seen in Fig. 8.

The algorithm of CMF is summarized in Algorithm 2.
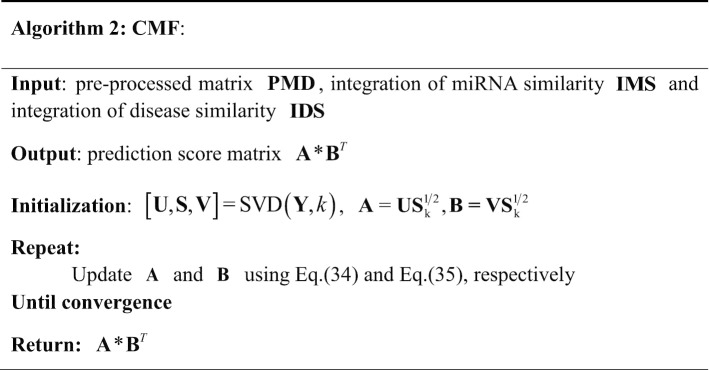


## Data Availability

The datasets that support the findings of this study are available in https://github.com/cuizhensdws.
